# Analysis of a mobile learning app for ophthalmology in Brazil

**DOI:** 10.5935/0004-2749.2023-0343

**Published:** 2024-08-30

**Authors:** Camila R. Koch, Rafael Scherer, Newton Kara Junior, Philipe Dourado Gripp, Alexandre Antonio Marques Rosa, Pedro Carlos Carricondo

**Affiliations:** 1 Universidade de São Paulo, São Paulo, SP, Brazil; 2 Hospital Humberto Castro Lima, Salvador, BA, Brazil; 3 Universidade Federal do Pará, Belém, PA, Brazil

**Keywords:** Ophthalmology, Mobile applications, Teaching, Smartphone, Education, distance, Internship and residency, Brazil

## Abstract

**Purpose:**

To determine and analyze the usability metrics of a free mobile learning app for ophthalmology in Brazil.

**Methods:**

Metric data from the management dashboard of the CBOQUIZ app were used. All users registered on the platform between March 2019 and June 30, 2021 were included. The number of questions answered, number of correct answers, number of questions answered and correct answers by subject area, and user performance by geographic region were analyzed.

**Results:**

There were 458 active users during the research period and 107,245 questions answered (average, 234.16 questions per user). Of the questions answered, 81,600 (75.5%) were correct and 2,645 were incorrect. The states in Brazil with the best performance were Espírito Santo, Paraiba, and Paraná. The subject area with the lowest hit rate was basic sciences (69.1%), within which embryology demonstrated the lowest hit rate (58.28%). The posterior segment had the highest number of questions answered, followed by miscellaneous topics and the anterior segment. Questions on strabismus were the least answered.

**Conclusion:**

The app was used consistently throughout the period studied, and participants adhered to this teaching modality. Performance asymmetry was observed across the Brazil states. The CBOQUIZ app can be used to homogenize ophthalmology teaching in the country.

## INTRODUCTION

Several institutions around the world have used distance learning alternatives in ophthalmology, especially after the 2019 coronavirus pandemic^(1,2)^. Knowledge acquisition and competence are essential components in the training of ophthalmologists. The use of new teaching tools, supported by technology such as mobile learning, is effective in this process.

The revolution in ophthalmology teaching methods during the pandemic demonstrated that interactive online classes increased the student participation from 15.6% to 46.7% after the pandemic^(1)^. A similar phenomenon was observed with several other types of digital teaching methods during the period. Furthermore, digital teaching in ophthalmology demonstrated a higher rate of satisfaction among undergraduate students and a greater ability to standardize teaching^(3,4)^.

According to the Pew Research Center^(5)^, Brazil is the fifth country with the highest cell phone usage (average 3 h/day), and it has the second largest app market. Thus, making a digital tool available to medical professionals aligns with this new reality. In this study, we have presented the analysis results of the O CBOQUIZ app, a free interactive gamified mobile learning application, which covers questions across various areas of ophthalmology with different levels of complexity.

## METHODS

This observational, analytical-descriptive, statistical study with quantitative reasoning, followed a transversal and retrospective approach. The study was conducted in line with the principles of the Declaration of Helsinki, Nuremberg Code, and National Health Council’s Norms for Research Involving Human Beings (Res. CNS 466/12). Informed consent was not required because no user identification data was used. Therefore, according to the regulations of the National Research Ethics Council, approval by the Research Ethics Council was not required.

### Main outcome measures

The deidentified metrics from the management dashboard of the CBOQUIZ app, which were made available by the Brazilian Council of Ophthalmology (*Conselho Brasileiro de Oftalmologia* – CBO), were used as a data source. The app has been available for download on Apple and Android platforms under the IDs 1,453,286,774 and *com.bredi.oftquiz*, respectively, since March 2019. The usability metrics of the ophthalmology teaching app in Brazil were studies to identify the overall performance of users and over time and the performance of users according to the Federation’s states and medical discipline.

### Exclusion and inclusion criteria

All users who registered on the platform between March 2019 and June 30, 2021 were included in the study. The registered users may not necessarily have been part of the CBO-accredited services. No individuals were excluded from the analysis.

### Disciplines analyzed

The disciplines were grouped into broad thematic areas as follows for analysis: basic sciences, which included anatomy, physiology, embryology, pharmacology, pathology, and semiology; miscellaneous, which included ocular oncology, iatrogenesis, ocular manifestations of systemic diseases, uveitis, and low vision; oculoplastic surgery, lacrimal ducts, and orbit; anterior segment, which included refractive surgery, eye bank, cataract, cornea, and contact lenses; and posterior segment, which included glaucoma, neuroophthalmology, retina, and vitreous.

### Statistical analysis

All computed data were organized in Google Sheets. Relative and absolute frequency measures as well as measures of central tendency and dispersion were calculated. The sample was not validated because a total population sampling method was utilized.

## RESULTS

A total of 458 active users were registered during the research period. According to their cell phone GPS, the users hailed from all the Federation’s states, with the exception of Roraima, Sergipe, Amapá, and Rondônia. Data such as sex and age were not recorded.

During the research period, 107,245 questions were answered by users, with an average of 189.1 questions per user. There were 81,600 correct answers (overall success rate, 76.08%) and 25,645 errors (Table 1).

**Table 1:** Total questions answered according to month during the study period (July 2019 to June 2021).

Months	Total	(%)
January	12,485	(11.6)
February	20,378	(19.0)
March	3,426	(3.2)
April	3,675	(3.4)
May	2,994	(2.8)
June	3,505	(3.3)
July	2,791	(2.6)
August	11,790	(11.0)
September	18,201	(17.0)
October	13,269	(12.4)
November	8,329	(7.8)
December	6,402	(6.0)
Total	107,245	-

**Figure 1:**
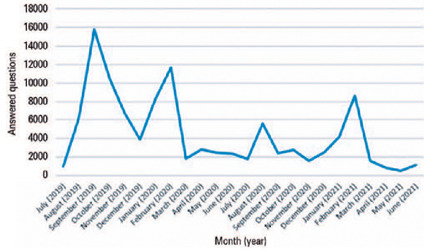
Monthly evolution of the general performance of CBOQUIZ users.

The app was used most between July 2019 and March 2020. There was an increase in the hit rate after July 2020 and in January 2020. The overall success rate was >70% in most of the months studied (Figure 1).

**Figure 2:**
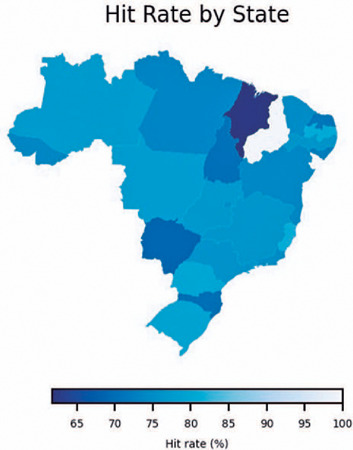
General performance of CBOQUIZ users in each Brazilian state.

There was considerable diversity among the Brazilian states in terms of user performance, with Paraná, Espirito Santo, and Paraíba exhibiting the highest success rates in the country (Figure 2).

Questions related to the posterior segment were answered more frequently than those of other disciplines, after mixed topics (n=25,787, 24%). The posterior segment topics included retinal and vitreous issues (hit rate, 81.4%), glaucoma (75.3%), and neuroophthalmology (67%). Of the three topics, neuroophthalmology questions demonstrated the lowest performance. The most accessed discipline was the “miscellaneous” category, with 26.456 questions answered (24.6%). The overall performance of subjects in the category was similar. The miscellaneous topics included uveitis (hit rate, 86.87%), ocular manifestations of systemic diseases (84%), ocular oncology (84.63%), iatrogenic diseases (82.49%), and low vision (77.7%). A total of 13,626 question on anterior segment were answered. The topics included contact lenses (accuracy rate, 82.47%), eye bank (77.28%), cataracts (74.27%), cornea (74.83%), and refractive surgery (65.17%). Among these topics, refractive surgery questions demonstrated the lowest performance, with a score well below the average of the other categories.

A total of 13.253 questions on “basic sciences” were answered, which included the following topics: “semiology” (hit rate, 90.89%), “anatomy” (72.31%), “pathology” (68.29%), “pharmacology” (66.81%), “physiology” (62.72%), and embryology (53.63%). The disciplines with the lowest number of questions answered were: “oculoplastic surgery”, “tear ducts”, and “orbits” (n=7,002; success rate, 75.79%; overall performance, similar); “optics and refraction” (n=4,321; success rate, 77.2%); and “strabismus“ (n=3,552; success rate, 74.26%).

## DISCUSSION

Ophthalmology is a specialty related to technological advances, which seeks to achieve lower costs and greater precision^(6)^. Furthermore, competency testing and knowledge acquisition are essential in the training of ophthalmologists. Thus, herein, we presented the analysis results of the CBOQUIZ app, an innovation that provides continued learning through technology. The general overview of knowledge and quality of educational services in the different Brazilian states, in addition to the percentage of correct answers per student in a given service, were generated by the app. Thus, CBOQUIZ helps improve teaching in ophthalmology, allows professionals to update themselves, reduces the devaluation of medical work, and helps generate specific proposals that improve each teaching service.

Hogarty et al. has highlighted how the smartphone has been the focus of several ophthalmological technologies^(7)^. CBOQUIZ is an app that offers a database of topics pertaining to the CBO and is organized by broad areas of knowledge with different levels of complexity. This interactive gamified app features countless daily tasks and resources for time-management. The app has cutting-edge design, game mechanics, and game-oriented intelligence in areas typically unrelated to ophthalmology. The need of a mere finger touch demonstrates its usability, and its accessibility anywhere at any given time demonstrates its practicality.

A candidate is evaluated via oral and written exams that categorize the candidates according to the scores^(8^, ^9,10)^. The act of measuring knowledge through tests is a prevalent practice, with the test score being a formal requirement of the educational system. Currently, the National Ophthalmology Test, “*Prova Nacional de Oftalmologia*”^(11)^ (PNO), is the main tool that evaluates an ophthalmology candidate’s knowledge in Brazil. Johnson et al.^(12)^ demonstrated that passing the annual exams of the “Ophthalmic Knowledge Assessment Program” (OKAP) is associated with a 5.43 times greater chance of passing the American Board of Ophthalmology Qualifying Exam (AAO-WQE). Furthermore, failing the three annual OKAP exams is associated with a nine times greater probability of failing the AAO-WQ exam. Completing questions from previous tests reportedly provides additional training. Thus, the CBOQUIZ may be an effective tool for preparing for the PNO.

The purpose of CBO’s progress test is to identify teaching flaws that need to be addressed before attempting the PNO. The use of evaluation tools allows the teaching methods and acquired knowledge to be assessed. The CBOQUIZ is another evaluation tool that can help identify issues, and in the face of observed difficulties, help plan corrective activities. Thus, late identification of potential failures can be avoided. Additionally, the knowledge demonstrated by the student’s responses reflect the efficacy of the teachers’ lectures. Thus, it helps indirectly evaluate the preceptors. Therefore, the CBOQUIZ app serves as a feedback of the teaching and learning process^(13)^. By generating a classification for the students, it also contributes to formative assessment. Because the CBOQUIZ app can identify learning issues, necessary knowledge points can be reinforced before the residents attempt the PNO and ophthalmological training can be analyzed.

In this study, we demonstrated that the overall performance was low in “basic sciences”, and the success rate was low in certain subspecialties, with performance asymmetry across states. Although topics on “ basic sciences” are not routinely experienced in the resident’s practice, they are the foundation of the Ophthalmologist’s professional life. Thus, there is a need for greater commitment of teaching services and students to learning basic sciences. Isolated subjects, such as neuroophthalmology and refractive surgery, had a low success rate because they require a higher degree of specialization, which may not be accessible to all the residents. This may justify the heterogeneity in the performance. Thus, policies to homogenize ophthalmology teaching in Brazil should be proposed. Among the four states without any users, only Sergipe has an accredited residency position in ophthalmology, with only one vacancy in 2019. This an important fact to note as residents are the platform’s largest target audience. Considering the minimum pass mark for the PNO, there was considerable diversity in performance between the states. Thus, this report may help establish minimum standards for ophthalmology teaching in the country.

The app was first launched in March 2019, which may justify the month’s low performance rate, as the target audience was still developing and familiarizing itself with the mechanics of the software. The overall success rates were within the range required to pass the PNO. This indicates that the quality of theoretical teaching covered in Brazil’s ophthalmology teaching services is adequate for passing the PNO. CBOQUIZ focuses on preparing students to attempt the exams, making it is a tool to evaluate learning. The analysis results aim to assist students in the teaching and learning process in order to achieve at least the minimum percentage score necessary to pass the PNO.

The CBOQUIZ application contributes to studying ophthalmology content for the selection process tests. It is a real-time evaluation tool for ophthalmology teaching across the country, which has been adapted to the current generation of students. The data generated can be used to establish skill-learning schedules in teaching services and customize reports for managers, which may improve the quality of ophthalmology training in the country. The application is currently active and being prepared in Spanish and English.
